# Homogeneity and Synchronous Dynamics of Microbial Communities in Particulate Biofilms: from Major Populations to Minor Groups

**DOI:** 10.1264/jsme2.ME11264

**Published:** 2011-12-01

**Authors:** Gaëlle Gévaudan, Jérôme Hamelin, Patrick Dabert, Jean-Jacques Godon, Nicolas Bernet

**Affiliations:** 1 INRA, UR50, Laboratoire de Biotechnologie de l’Environnement, Avenue des Etangs, Narbonne, F-11100, France

**Keywords:** CE-SSCP, molecular fingerprint, particulate biofilm, qPCR, triphasic bioreactor

## Abstract

Natural or engineered microbial populations often show variations over time. These variations may be due to environmental fluctuations or intrinsic factors. Thus, studying the dynamics of microbial diversity for different communities living in a spatially homogeneous landscape is of interest. As a model ecosystem, nitrifying biofilm communities were grown in a two litre inverse turbulent bed reactor (ITBR) containing an estimated 200 million small particles (about 150 μm in diameter). Each particulate biofilm is considered as a distinct community growing in the neighborhood of other similar particles, in a homogeneous and well-controlled environmental context. A molecular approach was adopted to test how microbial community structures might evolve: either in synchrony, converging or diverging. The shape of biofilm was observed by microscopy for each particle. The biomass content was evaluated by quantitative PCR and showed similar values for each particle. The microbial community structure was evaluated by Capillary Electrophoresis-Single Strand Conformation Polymorphism (CE-SSCP) fingerprinting and showed extraordinary homogeneity between particles, even though transitory community structures were observed when reactor operating conditions were modified. This homogeneity was observed for the *Bacteria* primer set but, more interestingly, was also observed when minor non-nitrifying bacteria making up the biofilm, representing about 5% and 10% of total cells, were targeted.

Laboratory-based microbial model systems have been shown to be useful in addressing ecological questions (3, 11). In particular, the behavior of replicated communities evolving in distinct ecosystems with similar environmental conditions may diverge over time (4, 7, 23, 32). The make-up of the microbial communities results from the balance of two opposite shaping forces, one leading to convergence while the other to divergence. Convergence of microbial communities can be attributed to environmental forces such as the rate of microbial population exchange and homogenous environmental parameters. Divergence of microbial communities can be attributed to various biotic forces, such as foundation events, predation, a heterogeneous structure within mature biofilm, etc. as observed in spatially separated replicated reactors (13) or local conditions in a spatially heterogeneous environment (18). The inverse turbulent bed reactor (ITBR) is a process which enables microbial biofilms to grow on millions of small silica particles. One biofilm on a silica sphere can be considered as a minute microbial community physically isolated from the others, but interacting with the other particles during mixing. ITBR technology has been successfully applied on a laboratory scale for nitrification (1, 2). This process permits bacterial colonization homogeneously on a biologically inert but mobile support (21). It is therefore especially suited for microbial ecology studies because environmental parameters of the whole ecosystem are easily adjusted (temperature, pH, dissolved oxygen concentration, substrate concentration and/or loading rate, etc.). Moreover, it is possible to monitor on-line substrate consumption and/or product accumulation; sampling is easy and can be automated.

The question addressed in this paper is: to what extent the dynamics of diversity is comparable for spatially distinct communities living in a homogeneous landscape? The ITBR technology was chosen as it is very effective for homogenizing silica beads, ensuring maximal exchange between biofilms on different beads. The nitrifying ecological process was chosen as a model system on account of its low functional diversity and high functional instability (2, 9). Particulate biofilms in a nitrifying ITBR were regularly characterized throughout the two-year experiment (2). Surprisingly, not only bacteria involved in nitrogen transformation through nitrification were found but much larger diversity emerged within the two-year-old biofilm, as sequences from putative anaerobic bacteria were recovered in this aerobic reactor (2). In the present study, different bacterial clusters not involved in nitrification were tracked (*Clostridia*- and *Lactobacillus*-like bacteria), both in terms of abundance (using quantitative PCR) and community structure (using CE-SSCP) (17). These clusters have been chosen because, as anaerobes and heterotrophic, they are not supposed to be dominant in a nitrification process fed with a mineral medium.

To check the heterogeneity within the whole ITBR, the differences in bacterial composition between individual silica spheres were assessed on four sampling dates.

Within the current study, we took advantage of ITBR technology to illustrate the synchronicity of the behavior of microbial communities from 200 million of colonized particles in different periods according to the different functions of a nitrifying reactor.

## Materials and Methods

### Reactor design and operation

A laboratory-scale inverse turbulent bed reactor (ITBR), previously described, was used (1, 2). It consists of a section of tubular polyvinyl-chloride, 0.085 m internal diameter and 1.1 m long, with an active fluidized volume of 2.84 L (aerated part) filled to 20% of this active volume with micro-particles. These spherical particles have an average diameter of dp=147 μm and a density of ρ_s_=690 kg m^−3^. The total particle surface area available in the lab-scale reactor was 13.4 m^2^. This material, a natural residue (Extendosphere; PQ Hollosphere, England), is light, mineral and granular and composed mainly of silica and alumina. Given the solid fraction in the static bed (ɛ_s0_=0.59 in a volume V=0.568), the total number of particles (N) in the reactor can be estimated by the following equation as about 200 million:

$$N=\frac{V{ɛ}_{S0}}{\frac{4}{3}\pi \frac{{dp}^{3}}{8}}$$

**Fig. 1:**
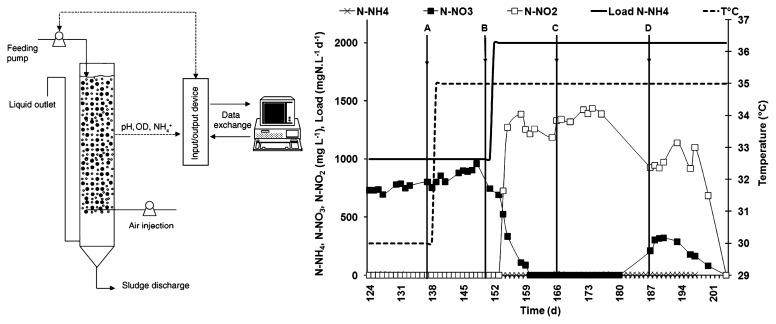
Experimental set-up of the laboratory-scale Inverse Turbulent Bed Reactor (ITBR) and experimental conditions. Arrows with letters A, B, C and D correspond to sampling dates with the associated operating conditions: (A) Load 1 gN L^−1^ d^−1^, 30°C and nitrate production, (B) Load 1 gN L^−1^ d^−1^, 35°C and nitrate production, (C) Load 2 gN L^−1^ d^−1^, 35°C and nitrite production, (D) Load 2 gN L^−1^ d^−1^, 35°C, both nitrate and nitrite production.

Air injection into the lower part, at a superficial velocity of 6 mm s^−1^, ensured aeration, mixing and particle fluidization. The temperature within the reactor was measured on-line and maintained at either 30 or 35°C by a water jacket (Fig. 1). pH was measured on-line using a InPro 4010 probe (Mettler Toledo, Greifensee, Switzerland) and maintained at 7.2 by the automatic addition of an alkaline solution (0.25 M KOH; 0.125 M Na_2_CO_3_). Dissolved oxygen concentration was measured with a 12/220 typeT probe (Mettler Toledo) connected to a dissolved oxygen transmitter 4300 (Mettler Toledo). Nitrate, nitrite and ammonium were analyzed off-line by an ion chromatography system (DIONEX 100) using conductivity detection.

Two liters of activated sludge from the municipal wastewater treatment plant in Coursan (Aude, France) at a concentration of 1.72 g VSS L^−1^ were used to inoculate the reactor after prior adaptation to the synthetic wastewater used as feed. The reactor was operated in batch mode with biomass recycling for 22 days, and then in continuous mode for 2.5 years. During this period, the reactor was fed with a synthetic mineral medium also containing a low concentration (353 g L^−1^) of complex organic carbon source (Viandox, Unilever, Rueil-Malmaison, France). After this long period, the reactor was fed with strictly mineral wastewater, described in Table 1, for 11 months in order to simplify the bacterial community. Figure 1 shows a schematic representation of the experimental ITBR used and the different operating conditions applied over time (increase of temperature from 30°C to 35°C on day 138 and a doubling of the ammonia nitrogen loading rate at 2 g-N L^−1^ d^−1^ on day 150) in the period of study. Four samplings were performed for biological analysis (Fig. 1), either just before any change in operating conditions (samplings A and B) or one week after any change in process performance (samplings C and D).

### Micro-particle sampling

Two forms of sampling were performed and analyzed on each sampling date. First, 1 mL samples (corresponding to about 350,000 colonized particles) were washed several times with 1X phosphate-buffered saline (PBS) (137 mM NaCl, 2.7 mM KCl, 4.3 mM Na_2_HPO_4_, 1.47 mM KH_2_PO_4_, pH 7.4) and then ground with an ice-cooled mortar in 2 mL 4 M guanidine thiocyanate in 0.1 M Tris-HCl (pH 7.5) and 600 μL of 10% N-lauroyl sarcosine. A 500 μL solution was immediately frozen and stored at −20°C before DNA extraction. The second type of sampling obtained single particles. Selected particles were washed with PBS and the level of colonization of individual particles was evaluated under a microscope (Olympus BX60, dark field, X40 magnification). Each selected particle was deposited in an amplification tube and ground in 5 μL 1X PBS and 100 μL Lyse-N-Go PCR reagent (Thermo Scientific, Rockford, IL, USA) with a Pasteur pipette. It was immediately frozen and stored at −20°C.

### DNA extraction

For the 1 mL samples, total genomic DNA was extracted and purified using a QiaAmp DNA stool mini kit, in accordance with the manufacturer’s instructions (Qiagen, Hilden, Germany). For single particle samples, 6 μL lysate was transferred into an amplification tube and submitted to thermal lysis. The thermocycling protocol for lysis was carried out according to the Lyse-N-Go manufacturer’s instructions.

### PCR amplifications

To analyze the structure of the entire bacterial community, the V3 region of the 16S rRNA gene was amplified using primers W49 and 5′-fluorescein phosphoramidite-labeled W104 (Table 2) (29). PCR amplifications were performed with a Mastercycler thermocycler (Eppendorf, Hamburg, Germany). The reaction mixtures contained 1×polymerase buffer, 0.2 mM dNTPs, 130 ng each primer, 0.5 U *Pfu* Turbo DNA polymerase (Stratagene, La Jolla, CA, USA), 1 μL genomic DNA, and water added to a final volume of 50 μL. The PCR conditions were as follows: an initial denaturation step for 2 min at 94°C followed by 25 cycles of a three-stage program with 30 s at 94°C, 30 s at 61°C, 30 s at 72°C, and final elongation for 10 min at 72°C. The reactions were stopped by cooling the mixture to 4°C. Amplification product sizes were confirmed by electrophoresis on a 2% (w/v) agarose gel.

The analyses of non-dominant bacterial clusters were performed after nested PCR (17). The first PCR step ensured the specificity of the clusters with either primer W108 or W109 paired with the W18 bacterial primer (Table 2). Primer W108, which targets 72% of the sequences belonging to the *Streptococcus-Lactobacillus-Enterococus* cluster and 24% of the sequences belonging to *Bacillus-Staphylococcus* cluster in the Ribosomal Database Project II, defined the *BSL* cluster. Primer W109, which targets 92% of the sequences belonging to the *Clostridium-Lachnospira-Eubacterium* cluster in the Ribosomal Database Project II, defined the *EC* cluster. PCR reactions contained about 100 ng sample genomic DNA (1 μL total DNA or 6 μL particle lysate), 5 μL AccuPrime *Taq* polymerase buffer, 100 ng of each primer, 5 U AccuPrime *Taq* polymerase (Invitrogen, Carlsbad, CA, USA), and enough water to bring the volume to 50 μL. The PCR conditions were as follows: denaturation for 2 min at 94°C followed by 25 cycles of 30 s at 94°C, 30 s at the annealing temperature (47°C for W108 and 45°C for W109) and 90 s at 72°C. The reaction was stopped by cooling the mixture to 4°C. Amplification product sizes were confirmed by electrophoresis on a 0.7% agarose gel. The nested PCR targeted the variable V3 region of the total bacterial and specific groups 16S rRNA genes, enabling the PCR products to be analyzed through CE-SSCP, as described below.

### Capillary Electrophoresis-Single Strand Conformation Polymorphism (CE-SSCP)

One microliter of diluted PCR products was added to 18 μL formamide and 1 μL internal size standard Rox 400 HD (Applied Biosystems, Carlsbad, CA, USA) diluted ten times. The sample was then denatured for 5 min at 94°C and placed directly on ice for 5 min. CE-SSCP was performed using the ABI 3130 genetic analyzer (Applied Biosystems) equipped with four 50 cm capillary tubes filled with 5.6% of conformation analysis polymer (Applied Biosystems) in the corresponding buffer and 10% glycerol. The injection of DNA into capillaries required 5 kV during 3 s. Electrophoresis was carried out at 15 kV and 32°C for 30 min per sample. CE-SSCP raw data were exported into the easy-to-handle csv format using Chromagna shareware (developed by Dr. Mark J. Miller at the US National Institute of Health) and statistics were performed using SAFUM (31) and Matlab 6.5 software (MathWorks). Principal component analysis was carried out using R 2.9.0 (19).

### Real-time quantitative PCR assay conditions

Quantitative PCR reactions were prepared in 25 μL, using 96-well optical reaction plates (Applied Biosystems) and ABI Prism 7000 (Applied Biosystems). For all analyses, the following components were added: 12.5 μL of qPCR Mastermix Plus for probes (Eurogentec, Liége, Belgium), 5 μL DNA extracts diluted 10 times or 6 μL particle lysate diluted 50 or 100 times, the forward primer, reverse primer and the TaqMan probe or the TaqMan MGB probe at appropriate concentrations (Table 2). TaqMan quantitative PCR using the MGB (minor groove binder) function ensures a low background signal and lowers probe Tm to be compatible with qPCR, thus avoiding mismatches. The cluster-specific primers used were the same as those for PCR amplification (Table 2). The working temperatures varied according to the bacterial cluster targeted. The temperatures used were 56°C for all bacteria using W102 and W105 primers with the W101 TaqMan probe; 57.5°C for the *BSL* cluster using W06 and W44 primers with the W198 TaqMan MGB probe; 55°C for the *EC* cluster using W06 and W44 primers with the W195 TaqMan MGB probe.

One standard curve was generated in each assay, using 10-fold dilutions in sterilized water (Aguettant Laboratory, Lyon, France) of PCR products from environmental clones referenced in (25). Full-length 16S rRNA gene clones derived from (25) and assigned as *Bacteria*, *EC* and *BSL* clusters were used as standards for qPCR analyses. The initial DNA concentrations were quantified using the GeneQuant pro spectrophotometer (Amersham Biosciences, Piscataway, NJ, USA). Six measurements were obtained per sample for each primer set. The relative abundance of the *BSL* and *EC* clusters were computed by comparison with the bacterial abundance in the sample, using R 2.9.0 software (19). The average number of bacterial cells was estimated by dividing the average number of 16S rRNA gene copies by a factor of 4.1, following (12) and to the rrnBD (http://ribosome.mmg.msu.edu/rrndb/).

## Results

### Reactor operation

During the period of study, different conditions were applied (Fig. 1): increase of temperature from 30°C to 35°C, doubling the ammonia loading rate applied to the reactor. As a consequence, first nitrate and then nitrite was produced by the nitrifying ecosystem. Indeed, an increase of the temperature coupled to an increase of the loading rate in a nitrifying reactor have been shown to favor ammonium-oxidizing bacteria (AOB) and strongly affect nitrite-oxidizing bacteria, and therefore to cause nitrite accumulation. From the four samples analyzed in this study, two were sampled during the nitrate production period (samplings A and B), and two during the nitrite production period (samplings C and D).

### Quantification of Bacteria and the BSL and EC clusters

**Fig. 2:**
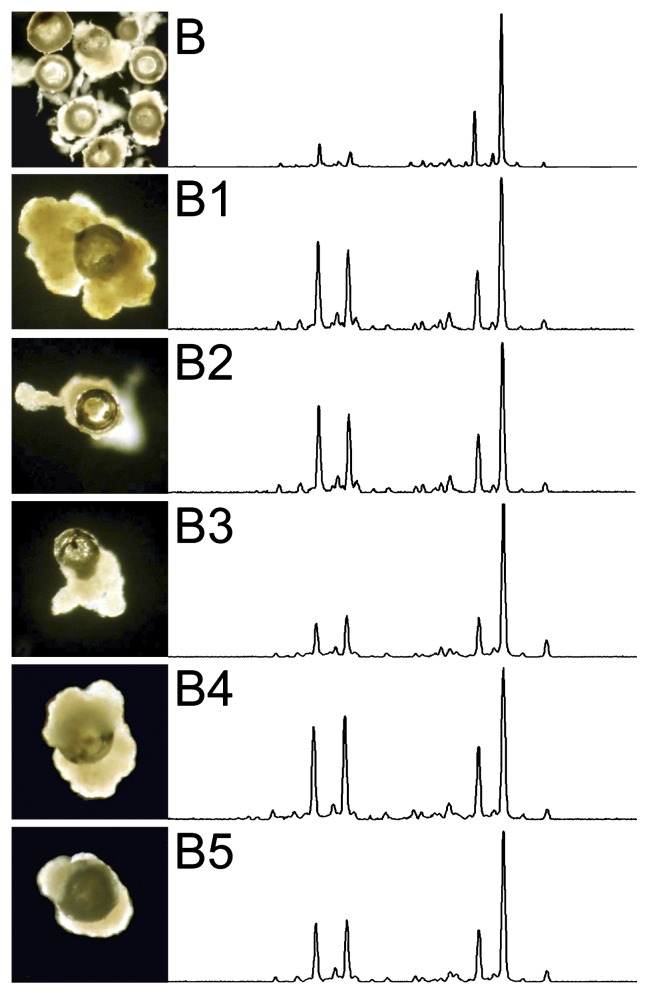
Images of colonized particles after 150 days of reactor operation (Series B) and their corresponding CE-SSCP fingerprinting profiles. The first row corresponds to about 350,000 particles (1 mL sample). The last five rows correspond to individual particles selected manually under the microscope.

The particles were steadily colonized over time, whatever the conditions applied to the reactor, as measured by the average number of 7.0×10^6^ bacterial 16S rRNA gene copies per particle (Table 3). It should be noted that visual inspection of individual particles under the microscope (Fig. 2) may lead to the erroneous conclusion that, because shapes differ, particles do not have similar bacterial density (Table 3).

*BSL* (*Bacillus-Streptococcus-Lactobacillus*) and *EC* (*Clostridium-Eubacterium*) bacteria could be detected in all the samples tested. The lower detection level was about ten 16S rRNA gene copies for both bacterial cluster-specific detection systems (Table 2) and good yields during quantitative PCR were obtained, varying between 95% and 98%. The relative proportions of these clusters in comparison to *Bacteria* abundance are presented for all sampling dates in Table 3. On average, the *BSL* cluster represented 18.5% of the bacterial 16S rRNA gene copies compared to 8.9% for the *EC* cluster. As *BSL* and *EC* bacteria harbor about twice as many 16S rRNA gene copies per cell as other bacteria, *BSL* and *EC* cells represented 9.3% and 4.5% of the biofilm cells, respectively. *Bacteroides* quantitative PCR was also applied to the same samples but erratic results were obtained (data not shown). This may indicate that such bacteria were only marginally present in our experimental system; however, their abundance was below the qPCR detection threshold.

Thus, three different colonizations, in terms of the proportion of the total biomass and phyla, could be simultaneously observed, and lead on to a study of the homogeneity of the microbial communities making up the biofilm.

### Homogeneous biofilm colonization among particles at a sampling date

Interestingly, the structure of the bacterial community (CE-SSCP profile) of one particle is very similar to that of a 1 mL sample containing 3.5×10^5^ particles (Fig. 2). CE-SSCP bacterial fingerprinting profiles were simple. A larger number of peaks was observed using the bacterial primers, with an average±SD of 19.7±3.7 peaks per profile and the constant dominance of 2 to 4 peaks. As expected, less diverse CE-SSCP profiles were obtained using primers targeting *BSL* and *EC* clusters, with 8.9±2.6 and 11.9±2.4 peaks, respectively.

**Fig. 3:**
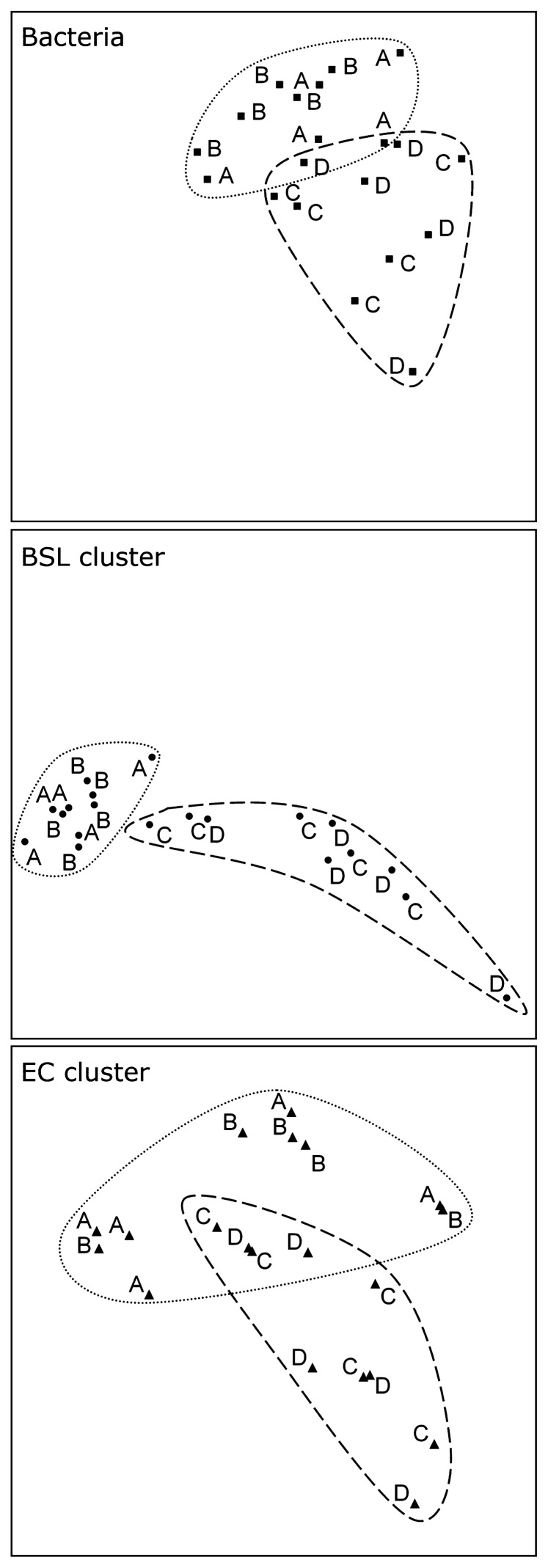
Principal Component Analysis (PCA) of 60 CE-SSCP fingerprinting profiles from individual particles. Letters refer to the sampling time given in Fig. 1. The fingerprints obtained separately from *Bacteria*, the *BSL* cluster and the *EC* cluster were statistically analyzed together but were displayed separately to avoid overcrowding the figure. Data from series A and B and C and D are circled as they correspond to similar functioning. A total of 62.1% of the variance is displayed in the biplot (39.9% for the horizontal axis and 22.2% for the vertical axis).

The overall genetic distance between particles, measured as the mean Euclidean distance between CE-SSCP profiles, was as low as 0.068 for the bacterial profiles and did not differ significantly for the *EC* or *BSL* bacterial clusters. Interestingly, the Euclidean distance between sampling times (*e.g.*, A–B, B–C, C–D) remained stable around 0.076±0.018 throughout the experiment for each of the primer sets. It should be noted that, for a given sampling date, the least abundant *EC* cluster (Table 3) also harbors the most heterogeneous communities among different particles. This can be easily observed by comparing the genetic distance between points in Fig. 3 that are directly related to the genetic distance of bacteria belonging to the *EC* cluster (Fig. 3, lower panel).

### Simultaneous evolution of individual particles over time: a similar fate

This homogeneity in the community structures of the colonized particles remained, including in the transient phases during which synchronous evolution of the molecular fingerprints was observed on the different particles analyzed (Fig. 3). This observation is valid not only for the major species represented as the major peaks on the bacteria profiles, but also for the minor clusters. The temperature increase (between samplings A and B) induced less variation than the increase of the ammonia nitrogen loading rate (between samplings B and C). Two distinct clusters of genetic profiles can be easily distinguished (circled in Fig. 3), whatever the bacterial cluster targeted. This is in agreement with the fact that the temperature increase did not modify the process, whereas an increase in the N loading rate completely inhibited the oxidation of nitrite to nitrate (Fig. 1).

## Discussion

A laboratory-scale nitrification bioreactor containing some 200 million colonized particles was used to test to what degree each particle bacterial community represented the others. The experimental set-up used can be seen as 200 million microbial communities structured in biofilm, with potential population exchange due to particle attrition. In this experimental ecosystem, the bacterial growth is counterbalanced by the erosion of particles from the biofilm through mixing, as in all particulate biofilm reactors (16). The equilibrium observed corresponds to different operating conditions. Thus, this experimental set-up provides a very convenient model for testing the dynamics of the diversity from small-size communities within a much larger community (3, 11).

It must be emphasized that the reactor was mainly operated under non-steady-state conditions, at least for the microorganisms. Indeed, in this kind of process, even if the reactor is operated at a quite short hydraulic retention time of only a few hours, the average biomass retention time is much higher because of the biomass retention as a biofilm.

The results based on CE-SSCP fingerprinting analysis show surprisingly that each colonized particle seems to be similar to the majority of the 200 million others in terms of the structure of the microbial community (Fig. 2). This was also true for abundance, with a constant colonization of around 10^6^ cells per particle (Table 2). Moreover, each single-particle community appears to be similar to the metacommunity constituted by the whole reactor (Fig. 2). The dynamics of the bacterial community observed on the scale of the whole reactor also held true on the individual-particle scale (Fig. 3). This similarity between community structure and the abundance of biofilm from different particles was observed at the dominance level, looking at all the *Bacteria*, but also at lower levels through *BSL* and *EC* clusters which, depending of the period, represent between 6.2 and 25.3% of bacterial sequences. The presence and even dominance of heterotrophic bacteria in nitrification reactors have been shown previously in suspended biomass systems (4, 5, 30). It is therefore not surprising to obtain significant concentrations of *BSL* and *EC* bacteria in a biofilm system, which is more heterogeneous in terms of the environmental conditions, including concentrations in oxygen and organic carbon from bacterial lysis.

It is apparent that each bacterial community from a single particle cannot be strictly identical to its neighbors, due to stochastic immigration events, but significant differences could not be detected either by CE-SSCP or qPCR tools. In this experiment, this was even true for bacteria belonging to the *EC* cluster with low abundance, representing 4.5% of cells. Below this value, we can draw no conclusions about particle homogeneity. For example, an attempt to target a rarer bacterial sequences belonging to the *Bacteroides* cluster produced inconsistent results (data not shown).

The question of spatial heterogeneity in open environments has already been addressed at the microbial level in soil (10, 22) and in marine (15, 20, 24) environments. Heterogeneity was always observed for soil but contrasting results were obtained from marine environments. Soil structure is basically heterogeneous whereas in marine environments, heterogeneity of the microbial structure relies on the nature of organic matter. The strength of this study using ITBR lies in the use of an easily adjustable bioreactor, in which environmental conditions can be strictly controlled and easily modified. In particular, mixing by air injection (Fig. 1) ensures good mixing conditions throughout the whole reactor, not only for the liquid but also for the solid phase, with a constant rate of particle attrition (21). If each particle is considered an individual ecosystem in ITBR, the bacterial community composition may change independently over time, as shown in replicated bioreactors without a physical link (6, 7, 13, 23, 32). Thus, this question of heterogeneity or homogeneity in a homogeneous environment was never a central concern in previous studies.

The make-up of the particle microbial communities observed results from the balance of two opposite shaping forces, one leading to convergence while the other to divergence. Convergence of microbial communities can be attributed to abiotic forces such as the exchange of microbial biomass due to attrition between particles, detritus released from mature biofilm and various biotic pressures such as predation, mutualism, etc. As previously observed, the microbial composition shows that this typically autotrophic and aerobic ecosystem within a 100 to 200 μm-thin biofilm also contains niches for heterotrophic organisms (*BSL* cluster) and anaerobic organisms (*EC* cluster) (2). Cell lysis due to cell death, virus infection or protozoa grazing may have released organic carbon, while other nutrients in the media permitted the build up of a complex ecosystem with heterotrophic organisms along with primary autotrophic nitrification activity. Divergence of microbial communities over time can be attributed to the growth of biofilm on each individual particle. In the second part of the study, when nutrient load had doubled (from day 150), the genetic heterogeneity between individual particles increased slightly (Fig. 3). Interestingly, microscope images (Fig. 1) show variable growth shapes of biofilm around the particles. In this experimental set-up, the convergence forces outclassed divergence forces throughout the experiment, but the higher growth rate of the biofilm when nutrient load was increased in the second part of experiment increased the heterogeneity of the system.

In conclusion, in a reactor with the same prevailing environmental parameters and with possible population exchange, each minute particle (150 μm in diameter), which corresponds to a colony-size scale (of about 10^6^ cells) can be studied individually. Each minute particle acts as a complex ecosystem similar to the meta-ecosystem on the reactor scale. This similarity between 200 million interacting island ecosystems could be observed for both the abundance and diversity of dominant and sub-dominant bacteria. Moreover, these ecosystems displayed synchronized behavior patterns over time. On this basis, one particle can be considered a representative sample of the whole reactor. This intuitive result was experimentally shown for the first time. Apart the new insight brought about microbial ecology, these results may well have interesting consequences for sampling strategies for well-mixed environments (*e.g.* flocs in wastewater treatment plants).

## Figures and Tables

**Table 1:** Composition of synthetic wastewater (250 mg N L^−1^)

Synthetic wastewater		Mineral medium	

Compounds	Concentrations	Compounds	Concentrations (g L^−1^)
NH_4_Cl (g L^−1^)	0.954	CaCl_2_·2H_2_O	7.34
K_2_HPO_4_ (g L^−1^)	0.35	MgCl_2_·6H_2_O	25.07
Na_2_HPO_4_ (g L^−1^)	0.35	FeCl_3_·6H_2_O	4.8
KHCO_3_ (g L^−1^)	0.2	MnCl_2_·4H_2_O	1.03
Mineral medium (mL L^−1^)	0.25	ZnCl_2_·2H_2_O	0.01
		CuCl_2_·2H_2_O	0.112
		NaMoO_4_·2H_2_O	0.0025

**Table 2:** Sequences and target positions of the primers used in this study

Primer name	Sequence (5′-3′)	*E. coli* position	Forward (F) Reverse (R)	Targeted 16S rRNA gene	Reference
W18	GAG TTT GAT CMT GGC TCA G	9	F	*Bacteria*	(8)
W108	ATT YCA CCG CTA CAC ATG	679	R	*BSL*	(28)
W109	CCC TTT ACA CCC AGT AA	561	R	*EC*	(27)
W104^a^	TTA CCG CGG CTG CTG GCA C^c^	533	R	*Universal*	(33)
W49	AGG TCC AGA CTC CTA CGG G	331	F	*Bacteria*	(33)
W06	CTAACTACGTGCCAGCAGC	507	F	*Bacteria*	This study
W44	TAC CRG GGT ATC TAA TCC	802	R	*Bacteria*	This study
W102	CGG TGA ATA CGT TCY CGG	1369	F	*Bacteria*	(26)
W105	GGW TAC CTT GTT ACG ACT T	1492	R	*Bacteria*	(26)
W101^b^	CTT GTA CAC ACC GCC CGT C	1389	F	*Bacteria*	(26)
W198^c^	CAT GTG TAG CGG TGR AAT	662	F	*BSL*	This study
W195^c^	CCC TTT ACA CCC AGT AA	561	R	*EC*	This study

a 5′ labeled with the fluorophore 6-carboxy-fluorescein (FAM)

b TaqMan probe labeled with 6-carboxy-fluorescein (FAM) as a reporter in 5′ and 6-carboxy-tetramethyl-rhodamine (TAMRA) as a quencher in 3′.

c TaqMan MGB probe (14)

**Table 3:** 16S rRNA gene bacterial gene abundance obtained by quantitative PCR for five particles at four sampling dates. The relative proportions of *BSL* and *EC* clusters are also shown, with the standard deviation in brackets.

Sampling dates	Bacteria	*BSL* cluster	*EC* cluster

Mean of 5 particles	16S rRNA gene copies (SD)	% of *Bacteria* (SD)	% of *Bacteria* (SD)
Series A	8.7×10^6^ (1.1)	7.5% (0.6)	6.2% (0.3)
Series B	3.0×10^6^ (0.2)	25.3% (0.4)	9.9% (0.4)
Series C	5.4×10^6^ (1.0)	21.7% (0.6)	9.2% (0.7)
Series D	11.0×10^6^ (6.1)	19.6% (0.5)	10.4% (0.5)
